# Predictors of Playing Augmented Reality Mobile Games While Walking Based on the Theory of Planned Behavior: Web-Based Survey

**DOI:** 10.2196/mhealth.8470

**Published:** 2017-12-11

**Authors:** Hyeseung Elizabeth Koh, Jeeyun Oh, Michael Mackert

**Affiliations:** ^1^ The Center for Health Communication Moody College of Communication The University of Texas at Austin Austin, TX United States; ^2^ Stan Richards School of Advertising and Public Relations Moody College of Communication The University of Texas at Austin Austin, TX United States; ^3^ Department of Population Health Dell Medical School The University of Texas at Austin Austin, TX United States; ^4^ School of Public Health The University of Texas Health Science Center at Houston Houston, TX United States

**Keywords:** mobile phone, pedestrians, safety on the street, psychological models, predictive value of tests, intention, age factors, attitude, social norms, self-efficacy, habits, immersion, self-report

## Abstract

**Background:**

There has been a sharp increase in the number of pedestrians injured while using a mobile phone, but little research has been conducted to explain how and why people use mobile devices while walking. Therefore, we conducted a survey study to explicate the motivations of mobile phone use while walking

**Objective:**

The purpose of this study was to identify the critical predictors of behavioral intention to play a popular mobile game, *Pokemon Go,* while walking, based on the theory of planned behavior (TPB). In addition to the three components of TPB, automaticity, immersion, and enjoyment were added to the model. This study is a theory-based investigation that explores the underlying mechanisms of mobile phone use while walking focusing on a mobile game behavior.

**Methods:**

Participants were recruited from a university (study 1; N=262) and Amazon Mechanical Turk (MTurk) (study 2; N=197) in the United States. Participants completed a Web-based questionnaire, which included measures of attitude, subjective norms, perceived behavioral control (PBC), automaticity, immersion, and enjoyment. Participants also answered questions regarding demographic items.

**Results:**

Hierarchical regression analyses were conducted to examine hypotheses. The model we tested explained about 41% (study 1) and 63% (study 2) of people’s intention to play *Pokemon Go* while walking. The following 3 TPB variables were significant predictors of intention to play *Pokemon Go* while walking in study 1 and study 2: attitude (*P*<.001), subjective norms (*P<*.001), and PBC (*P=*.007 in study 1; *P<*.001 in study 2). Automaticity tendency (*P<*.001), immersion (*P=*.02), and enjoyment (*P=*.04) were significant predictors in study 1, whereas enjoyment was the only significant predictor in study 2 (*P=*.01).

**Conclusions:**

Findings from this study demonstrated the utility of TPB in predicting a new behavioral domain—mobile use while walking. To sum up, younger users who are habitual, impulsive, and less immersed players are more likely to intend to play a mobile game while walking.

## Introduction

### The Ubiquity of Mobile Devices

Despite the efficiency and convenience of communication technology, the increased use of mobile media technology produces alarming safety issues in society, such as mobile phone use while driving [1] and walking [2,3]. Although researchers have been paying a great deal of attention to the perils of mobile phone use while driving [1], concerns about using a mobile phone while walking have only recently been raised. Researchers have examined the effects of mobile phone usage on cognitive and gait performance [4-6]. Their findings indicated that people who used mobile phones while walking were cognitively distracted and did not pay enough attention to their surroundings, such as traffic signals or oncoming automobiles. Pedestrians who use their mobile phones while walking cannot maintain their balance, which may cause an increase in unsafe behaviors, injuries, or even death.

Despite the prevalence and popularity of mobile device use in everyday life across all age groups, there has been little interest in exploring and understanding what motivates people to use mobile devices, such as a mobile phone, while walking. This study attempts to fill in this gap in prior literature in the psychology of mobile device use. The findings from this study will provide a better understanding of people’s mobile device use in everyday life, which may help health communication researchers and practitioners to create interventions targeting risky behaviors related to mobile device use. The next section provides a short review of mobile phone use literature and the theory of planned behavior (TPB) literature.

### Prior Work on Perils of Mobile Phone Use While Walking

There is extensive research examining the effects of mobile phone use on distracted driving [1,7]. Recently, attention has shifted to the dangers of texting while walking [2,6,8]. Similar to the harmful impact of texting while driving for drivers, the dual task of texting while walking visually and cognitively distracts pedestrians from the road [4]. Several studies examined the effect of mobile phone use while walking on pedestrians’ gait performance. For example, Lamberg and Muratori [9] found that people who used a mobile phone while walking demonstrated a reduced walking speed and greater lateral deviation from a straight path than people who did not use a mobile phone while walking. Furthermore, people texting while walking showed greater interference effects from mobile phone use than those talking on the phone while walking. These findings imply that a dual task such as texting or playing games on a phone while walking would be a factor that threatens pedestrians’ safety by increasing cognitive demand or limiting availability of visual information. These findings also raise concerns about pedestrian safety due to mobile phone use while walking in general. Furthermore, researchers have shown a significant association between mobile phone use while walking and the risk of collisions, falls, and traffic accidents [2,10].

The pedestrian safety issue, playing augmented reality mobile games such as *Pokemon Go* while walking [11,12], has been recently highlighted by media. *Pokemon Go* has been downloaded more than 550 million times and continues to be downloaded worldwide [13], and Wagner-Greene et al indicated that playing *Pokemon Go* could lead to unsafe walking without paying attention to surroundings [12]. Although some research found a positive effect of playing *Pokemon Go* on the amount of daily physical activity [12,14], the findings from recent research are contradictory at best. For example, Howe et al found that the increase in the average daily steps was significant only in the first week, eventually decreasing over the following 5 weeks [15]. There was no significant difference in the average daily steps between *Pokemon Go* players and nonplayers 6 weeks after beginning the game.

Although the potential health benefits of playing *Pokemon Go* are controversial, the negative aspects of playing *Pokemon Go* while walking are potentially life threatening. Health experts reported increased user injuries because of *Pokemon Go* and warn against the risks associated with playing augmented reality games while walking [10,16]. To prevent the potentially severe consequences due to unsafe mobile use practices including augmented reality gaming while walking, it is necessary to identify significant factors associated with mobile use while walking to inform people of the potential risks.

### Theoretical Background: The Theory of Planned Behavior

This study uses TPB to find salient predictors of people’s intentions to play a mobile game, in particular *Pokemon Go*, while walking. TPB has been applied to understand distracted driving among people, specifically understanding the factors that lead people to use mobile phones while driving [1,7]. TPB posits that an individual is willing to choose to perform or not to perform a certain behavior through rational considerations [17]. The theory of reasoned action (TRA) [18] and TPB [17] assume that individuals systematically use and evaluate available information about outcomes associated with behaviors before engaging in the behaviors. In addition, those volitional behaviors can be best predicted from a person’s willingness or behavioral intention, which is defined as “a measure of the likelihood that a person will engage in a given behavior” [19].

According to TRA, a person’s behavioral intention can be predicted from one’s attitude toward the performance of the behavior and subjective norms. Within the TRA framework, attitude toward a behavior is defined as the extent to which a person evaluates a certain behavior favorably or unfavorably, and subjective norms refer to a person’s perceived social pressure from important others (eg, family members or close friends) about whether one should carry out the behavior or not [17]. Both attitude and subjective norms contain a belief and evaluative component. Later, TPB added a component, perceived behavioral control (PBC), to predict a person’s behavioral intention. PBC refers to the degree of control that individuals perceive themselves to have over performance of a behavior, which has a direct impact on a behavior. TPB has been widely applied to various research topics including health-related behaviors [20-22], binge drinking [23], recycling [24], organ donation [25,26], and distracted driving [1,7,27] to predict and explain human behavior. Findings from previous studies indicated that TPB has a predictive and explanatory power [20].

Thus, this study hypothesizes that positive attitudes toward mobile game play (H1), greater perceived subjective norms (H2), and a higher level of PBC (H3) would lead to a strong intention to play mobile games while walking through cognitive evaluation:

H1: As people have more positive attitudes toward playing *Pokemon Go* while walking, they would more likely intend to play *Pokemon Go* while walking.

H2: As people perceive greater social pressure on playing *Pokemon Go* while walking, they would more likely intend to play *Pokemon Go* while walking.

H3: As people have a greater degree of PBC over playing *Pokemon Go* while walking, they would more likely intend to play *Pokemon Go* while walking.

Although previous findings have shown the effectiveness of TPB to predict and explain human behavior in many different contexts, researchers argue for the necessity to improve the predictive and explanatory power of the theory with the inclusion of relevant factors such as affective states (ie, anticipated regret), moral norms, or personal norms [28-30]. This study attempts to add relevant components to TPB considering the characteristics of the mobile game behavior in question.

### Additional Predictors in Mobile Communication

One of the most common criticisms on TPB has been that it only focused on the conscious, reasoned behavioral motivations [31]. TPB recognizes cases in which individuals sometimes less consciously use and evaluate available information before engaging in behaviors (ie, automaticity) [32]. People may not need to assess their beliefs and relevant information with great consciousness to decide whether they perform a behavior once they have performed it many times. They could retrieve their stored attitudes and intentions from memory and make decisions without much cognitive effort. Bayer et al argue that people rely on automatic (less conscious) or immersive (more conscious) behavioral orientations when they engage in communication activities using mobile devices [33]. Using mobile devices is a behavior that is performed in a less conscious manner (ie, with a high level of automaticity) or in a high conscious manner (ie, with a high level of immersion), depending on the context or personality (ie, trait self-regulation or trait mindfulness) [33]. For example, people sometimes use mobile devices for calling, texting, surfing the Web, or playing mobile games with minimal consciousness (eg, automaticity, habits, and impulses), whereas they use mobile devices for the same behaviors with conscious effort other times (eg, flow, immersion, presence, and absorption). That is to say, mobile communication involves both less conscious (ie, automaticity) and more conscious (ie, immersion) processes based on the levels of media users’ perceived behavioral consciousness.

Bayer et al empirically tested the distinct roles of automaticity and immersion in mobile communication [33]. In their study, automaticity refers to a behavioral process in mobile device use including the following four behavioral dimensions: lack of attention, lack of awareness, lack of intention, and lack of control. Immersion refers to a behavioral process in mobile device use with maximal behavioral attention and awareness. Immersive process also includes a lack of temporal and spatial awareness because of the great concentration on the media activities. The interactive nature of mobile communication such as texting or playing augmented reality games is associated with an immersive behavioral process such that users actively attend to their mobile-mediated interactions and construct a virtual social space while navigating the physical environment. Along the spectrum, automaticity lies on the less conscious end, whereas immersion lies on the more conscious end.

On the basis of the spectrum, Bayer et al (study 1) explored whether automaticity and immersion independently or simultaneously influence mobile use behaviors such as texting (ie, texting frequency and affective benefits of texting) [33]. They found that only automatic behavioral orientation (ie, less conscious use of texting) was positively associated with texting frequency, whereas both automatic and immersive behavioral orientations were positively related to perceived affective benefit of texting (called affective temptation). In other words, automatic behavioral orientations and immersive behavioral orientations would cowork when people feel that mobile use activity produces positive affect. Although automaticity and immersion are located on the opposite side of a conceptual continuum, both Bayer et al and other researchers have found that human behavior emerges from a combination of the unconscious and conscious processes [33,34].

Given that previous research found both automaticity and immersion as significant predictors of mobile use behavior, this research adds these two different behavioral tendencies to TPB, automaticity and immersion in mobile communication. Previous research recommends to simultaneously incorporate two mobile use related−behavioral orientations [33]. Thus, this study hypothesizes that greater automatic behavioral orientation (H4) and greater immersive behavioral orientation (H5) toward mobile game play would lead to a strong intention to play mobile games while walking:

H4: As people have greater automaticity of playing *Pokemon Go* while walking, they would more likely intend to play *Pokemon Go* while walking.

H5: As people have greater immersion in playing *Pokemon Go* while walking, they would more likely intend to play *Pokemon Go* while walking.

Another factor relevant to playing a mobile game while walking, in general, is an individual’s feeling of enjoyment [35]. Such an intrinsic motivation encourages people to persist in performing a behavior [36]. Prior literature found that enjoyment was a significant predictor of intention to play Web-based games [35,37]. Thus, this study assesses the effect of an individual’s enjoyment on their decision to play a mobile game while walking.

H6: As people experience greater enjoyment of playing *Pokemon Go* while walking, they would more likely intend to play *Pokemon Go* while walking.

This paper addresses this emerging health issue related to mobile communication by testing 2 samples. Study 1 used a sample of young college students aged 18 to 34 years. Study 2 addressed the lack of diversity of the sample in study 1 by recruiting a sample of people aged 18 to 65 years from Amazon Mechanical Turk (MTurk). The following section details the methodology for this study.

## Methods

### Participants and Procedure

This study used a convenience sample and Web-based survey to ask participants to report their intention of playing *Pokemon Go* while walking, automaticity tendency, immersion tendency, enjoyment, and 3 TPB components, including attitude, subjective norms, and PBC. It took about 15 minutes to complete the Web-based survey. This study was approved by an Institutional Review Board and pretested by researchers.

#### Study 1

Participants were recruited online from a nonprobability sample (ie, the general communication pool) at a large southern university in the United States from November 2016 to April 2017. Participants were required to be mobile phone users to participate in the complete survey of the psychological processes. In recognition of their participation, they received extra course credit.

#### Study 2

As student convenience samples have limited generalizability, the Web-based sample from MTurk was utilized to provide a more diverse sample for this study [38-40]. This is particularly relevant because prior research has related mobile device use to age and life phase [33,41]. Participants were recruited from MTurk in the United States in March 2017. Only US workers could participate in the study to minimize the effect of different cultural background on the outcome variable. Participants were required to be mobile phone users to participate in the complete survey. Participants received US $ 0.75 in recognition of their participation.

### Measurement

The same measures were used in both study 1 and study 2. Each measure was checked for inter-item correlations, item contribution to scale reliability, and internal consistency. All measures were 7-point scales except for the automaticity and immersion, which used a 5-point scale anchored by 1 (*not at all*) and 5 (*completely*). All measures are available in Multimedia Appendix 1.

#### Attitude

Participants’ attitude toward playing *Pokemon Go* while walking was assessed with a 7-point semantic differential scale adopted from a previous study [42]. Participants were asked to respond to the statement “For me, playing *Pokemon Go* while walking would be...” The following items were included: beneficial/harmful, foolish/wise, unpleasant/pleasant, enjoyable/unenjoyable, bad/good, favorable/unfavorable, and positive/negative.

#### Subjective Norms

To measure the extent to which participants perceived behavioral expectation from their important people, seven items were used [1,43]. An example item is “Those people who are important to me would want me to play *Pokemon Go* while walking.” (1=strongly disagree, 7=strongly agree).

#### Perceived Behavioral Control

The extent to which participants perceived that they had control over playing *Pokemon Go* while walking was assessed using three items with a 7-point Likert-type scale adopted from a previous study [44]. An example item includes “How much personal control do you feel you have over playing *Pokemon Go* while walking?” (1=not at all, 7=very much so). To improve the reliability, an item that did not contribute to scale reliability was removed from the scale.

#### Automaticity

Automaticity was defined as a behavioral orientation that occurs without conscious awareness. Four items were used to measure the extent of participants’ automatic behavioral orientations toward playing *Pokemon Go* while walking. The items were adapted from a previous study [33]. Automaticity includes the following four dimensions: lack of behavioral attention, lack of behavioral awareness, lack of behavioral intention, and lack of behavioral control. An example item is “When I play *Pokemon Go*, I do it without thinking.”

#### Immersion

Immersion was defined as a behavioral orientation that occurs in a conscious manner. Four items were used to measure the extent of participants’ immersive behavioral orientations toward playing *Pokemon Go* while walking. Immersion includes the following four dimensions: maximal behavioral attention, maximal behavioral awareness, lack of spatial awareness, and lack of temporal awareness [33]. The items were adapted from a previous study [33]. An example item includes “When I play *Pokemon Go*, my eyes are fixed on doing it.”

#### Enjoyment

To assess individuals’ feeling of enjoyment while playing *Pokemon Go* while walking, four items were adopted from a previous study [35]. One of the examples includes “Playing *Pokemon Go* is enjoyable.” (1=strongly disagree, 7=strongly agree).

#### Intention to Play Pokemon Go While Walking

To measure behavioral intention to play *Pokemon Go* while walking, this study used three items adopted from previous research [1]. An example item is, “I plan to play *Pokemon Go* while walking.” (1=strongly disagree, 7=strongly agree). Tables 1 and 2 present descriptive statistics of the measures, including means, standard deviations (SDs), and Cronbach alphas for study 1 and study 2.

**Table 1:** Means, standard deviations, and Cronbach alphas for study 1 variables.

Study 1 variables	Mean (SD)	Cronbach alpha
Intention	3.90 (1.32)	.98
Attitude	3.91 (1.32)	.96
Subjective norms	3.86 (1.04)	.82
PBC^a^ (after deleting #3)	6.12 (1.01)	.71
Automaticity	2.09 (0.96)	.87
Immersion	2.37 (0.99)	.93
Enjoyment	4.84 (1.23)	.92

^a^PBC: perceived behavioral control.

**Table 2:** Means, standard deviations, and Cronbach alphas for study 2 variables.

Study 2 variables	Mean (SD)	Cronbach alpha
Intention	4.28 (1.98)	.97
Attitude	4.68 (1.43)	.97
Subjective norms	4.36 (1.42)	.91
PBC^a^ (after deleting #3)	6.18 (1.08)	.77
Automaticity	2.54 (1.10)	.89
Immersion	2.92 (1.05)	.91
Enjoyment	5.36 (1.33)	.91

^a^PBC: perceived behavioral control.

Finally, questions regarding demographic information, including age, gender, ethnicity, and mobile phone addiction, were measured.

### Analytic Plan

To test the study hypotheses, hierarchical regression analysis was employed. Before conducting hierarchical regression analyses, the categorical variable was dummy coded. For gender, male was coded as 0 and female was coded as 1. Other continuous variables, except the dependent variable, were mean centered to avoid potential multicollinearity [45]. Two demographic variables, age and gender, were included as covariates in the analyses based on the findings from prior literature [33,41,46]. For each analysis performed, the first block of the regression analyses contained the study covariates. The second block of the regression analyses contained the three TPB predictors, followed by additional predictors, automaticity, immersion, and enjoyment, in the third block. Tables 3 and 4 reported the zero-order correlation matrix of continuous variables for study 1 and study 2.

**Table 3:** Correlation matrix of variables in study 1 (N=262), shown as Pearson correlation coefficient r (*P* value).

Study 1 variables		1	2	3	4	5	6	7	8	9
1	Intention	—^a^								
2	Attitude	.53 (.001)	—							
3	Subjective norms	.50 (.001)	.57 (.001)	—						
4	Perceived behavioral control	−.11 (.08)	.09 (.14)	.05 (.41)	—					
5	Automaticity	.25 (.001)	.06 (.38)	.13 (.03)	−.15 (.02)	—				
6	Immersion	.13 (.03)	.13 (.04)	.14 (.02)	−.10 (.10)	.60 (.001)	—			
7	Enjoyment	.37 (.001)	.42 (.001)	.38 (.001)	−.02 (.79)	.14 (.03)	.22 (.001)	—		
8	Age	−.10 (.10)	.01 (.84)	.05 (.43)	−.02 (.79)	−.07 (.23)	−.01 (.94)	−.08 (.20)	—	
9	Gender	.01 (.91)	−.08 (.23)	.01 (.85)	−.03 (.62)	−.12 (.049)	−.09 (.17)	.12 (.05)	−.04 (.54)	—

^a^— signifies the correlation of 1.

**Table 4:** Correlation matrix of variables in study 2 (N=179), shown as Pearson correlation coefficient r (*P* value).

Study 2 variables		1	2	3	4	5	6	7	8	9
1	Intention	—^a^								
2	Attitude	.73 (.001)	—							
3	Subjective norms	.65 (.001)	.64 (.001)	—						
4	Perceived behavioral control	−.05 (.52)	.11 (.14)	.17 (.02)	—					
5	Automaticity	.27 (.001)	.18 (.02)	.24 (.001)	−.32 (.001)	—				
6	Immersion	.41 (.001)	.36 (.001)	.32 (.001)	−.05 (.49)	.63 (.001)	—			
7	Enjoyment	.51 (.001)	.56 (.001)	.42 (.001)	.24 (.001)	.19 (.01)	.43 (.001)	—		
8	Age	−.08 (.27)	−.09 (.23)	−.15 (.049)	.09 (.21)	−.15 (.04)	−.05 (.52)	.02 (.74)	—	
9	Gender	−.20 (.008)	−.12 (.12)	−.13 (.09)	.12 (.12)	−.32 (.001)	−.29 (.001)	−.03 (.68)	.08 (.26)	—

^a^— signifies the correlation of 1.

## Results

### Sample Characteristics

#### Study 1

A total of 417 participants participated in a Web-based survey. After removing individuals who played other mobile games that were not *Pokemon Go* (n=148) and individuals who missed reporting main predictors (n=7), a total of 262 participants who completed the Web-based survey were used to test hypotheses. The age of participants ranged from 18 to 34 years. The mean age of the participants was 20.32 (SD 1.86) years. Most participants were white (47.7%, 125/262), but the sample included people who reported as Asian/Asian American (28.6%, 75/262), Hispanic/Hispanic American (18.3%, 48/262), African American (2.3%, 6/262), and other (3.1%, 8/262). The others included participants who answered biracial (n=2), Middle Eastern (n=1), and mixed (n=2). The study sample included more females (63.7%, 167/262).

#### Study 2

A total of 264 participants participated in a Web-based survey. After removing individuals who played other mobile games that were not *Pokemon Go* (n=58) and individuals who missed reporting main predictors (n=27), a total of 179 participants who completed the Web-based survey were used to test hypotheses. The age of participants ranged from 18 to 65 years. The mean age of the participants was 30.61 (SD 9.04) years. Most participants were white (70.4%, 126/179), but the sample included people who reported as Hispanic American (10.1%, 18/179), Asian (8.9%, 16/179), African American (6.1%, 11/179), Native American (1.7%, 3/179), and other (2.8%, 5/179). Regarding participants’ highest education level, 90.5% (162/179) of participants had completed some college or a 2-year degree or higher. Finally, regarding income level, 30.7% (55/179) of the participants had an income of US $25,000 to US $49,999, followed by US $50,000 to US $74,999 (24.6%, 44/179), over US $100,000 (16.2%, 29/179), less than US $25,000 (15.6%, 28/179), and US $75,000 to US $94,999 (12.8%, 23/179). The study sample included more males (56.9%, 102/179).

### Hypothesis Tests

#### Study 1

The results show that the overall model, including all the predictors, was significant, *F*_8,253_=23.95, *P*<.001, adjusted *R*^2^=.41. In the first block, none of the covariates was a significant predictor of intention to play Pokemon Go while walking. However, age was a significant predictor in the second and third block. Older people were less likely to intend to play *Pokemon Go* while walking. To test the hypotheses 1 to 3, when the three main components of TPB were entered into the second block of the regression analysis, all three predictors were significant. As people had more positive attitudes (beta=.40, *P*<.001, semipartial correlation [*sr*]=.32) and perceived stronger social pressure from people around them (beta=.29, *P*<.001, *sr*=.23), they were more likely to play *Pokemon Go* while walking. Thus, the data were consistent with H1 and H2. Although PBC was a significant predictor of intention, the relationship was in the direction opposite to that hypothesized relationship. As people with lower levels of perceived control (beta=−.16, *P*=.002, *sr*=−.15), they were more likely to intend to play *Pokemon Go* while walking. Thus, the data were not consistent with H3.

Hypotheses 4 to 6 predicted that automaticity, immersion, and enjoyment would be significant predictors of intention to play *Pokemon Go* while walking, which would improve the predictive power of the model. When these additional three variables were entered into the third block, the regression coefficients of the three main components of TPB were readjusted, taking into account the three additional predictors entered in the third block. All three TPB components remained significant even after including the additional three variables. The additional three variables were significant, which increased the explained variances in intention by 4.4%. As people who perceived greater automatic behavioral orientation (beta=.24, *P<*.001, *sr*=.19) and felt greater enjoyment (beta=.11, *P=*.04, *sr*=.10), they were more likely to intent to play *Pokemon Go* while walking. Thus, the data were consistent with H4 and H6. That is, the results indicated that as people played *Pokemon Go* while walking in a less conscious manner and felt greater enjoyment, they were more likely to play *Pokemon Go* while walking. Although perceived immersive behavioral orientation was a significant predictor of intention, the relationship was in the direction opposite to that hypothesized relationship. As people perceived less immersive behavioral orientation (beta=−14, *P=*.02, *sr*=−.11), they were more likely to intend to play *Pokemon Go* while walking. Thus, the data were not consistent with H5. Table 5 reported regression analysis results for study 1.

#### Study 2

The overall model, including all the predictors, was significant, *F*_8,170_=38.76, *P=*.001, adjusted *R*^2^=.63. In the first block, gender was a significant predictor of intention to play *Pokemon Go* while walking (beta=−.19, *P=*.01, *sr*=−.19). Women were less likely to intend to play *Pokemon Go* while walking. However, none of the covariate was a significant predictor in the second and third block. When the three main components of TPB were entered into the second block of the regression analysis, attitude (beta=.53, *P<*.001, *sr*=.41) and subjective norms (beta=.33, *P<*.001, *sr*=.25) were statistically significant. PBC was a negative predictor of behavioral intention toward playing *Pokemon Go* while walking (beta=−.16, *P=*.001, *sr*=−.15). The results showed that the more positive attitudes, stronger subjective norms, and less perceived behavior control over playing *Pokemon Go* while walking individuals had, the more strongly they intended to play *Pokemon Go* while walking. Thus, the data were consistent with H1 and H2, but inconsistent with H3.

The same procedure that was used in study 1 was conducted to test the Hypotheses 4 to 6. All three TPB components remained significant even after including the additional three variables (see Table 6). Enjoyment (beta=.15, *P=*.01, *sr*=.12) was the only significant predictor of intention to play *Pokemon Go* while walking. An additional 2.2% of the variances in the behavioral intention was explained. Thus, it was concluded that the data were consistent with H6 but inconsistent with H4 and H5. The regression analysis results for study 2 are presented in Table 6.

**Table 5:** Regression results for intention to play a mobile game while walking in study 1 (N=262).

Predictors		*B* ^a^	Standard error (SE)	Beta	*t* (df=260)	*P* value	*sr* ^b^
**First block** ^c^							
	Age	−.10	.06	−.10	−1.66	.10	−.10
	Gender	.01	.23	.003	0.05	.96	.003
**Second block** ^d^							
	Attitude	.54	.08	.40	6.59	<.001	.32
	Subjective norms	.49	.10	.29	4.76	<.001	.23
	PBC^e^	−.27	.09	−.16	−3.15	.002	−.15
**Third block** ^f^							
	Attitude	.51	.08	.38	6.13	<.001	.29	
	Subjective norms	.41	.10	.24	4.06	<.001	.19	
	PBC^c^	−.23	.09	−.13	−2.74	.007	−.13	
	Automaticity	.44	.11	.24	3.92	<.001	.19	
	Immersion	−.25	.11	−.14	−2.31	.02	−.11	
	Enjoyment	.16	.08	.11	2.04	.04	.10	

^a^*B*: unstandardized coefficients.

^b^*sr*: semipartial correlation.

^c^*F*_2,259_=1.39, *P=*.25, adjusted *R*^2^=.003.

^d^*F*_change3,256_=52.31, *P<*.001, *R*^2^_change_=.38.

^e^PBC: perceived behavioral control.

^f^*F*_change3,253_=6.57, *P<*.001, *R*^2^_change_=.044.

**Table 6:** Regression results for intention to play a mobile game while walking in study 2 (N=197).

Predictors		*B* ^a^	Standard error (SE)	Beta	*t*	*P*	*sr* ^b^
**First block** ^c^							
	Age	−.02	.02	−.07	−0.91	.36	−.07
	Gender	−.76	.30	−.19	−2.59	.01	−.19
**Second block** ^d^							
	Attitude	.73	.08	.53	8.73	<.001	.41
	Subjective norms	.46	.09	.33	5.28	<.001	.25
	PBC^e^	−.29	.09	−.16	−3.29	.001	−.15
**Third block** ^f^							
	Attitude	.60	.09	.44	6.56	<.001	.30
	Subjective norms	.44	.09	.32	5.06	<.001	.23
	PBC	−.35	.10	−.19	−3.63	<.001	−.17
	Automaticity	−.06	.12	−.04	−0.54	.59	−.03
	Immersion	.14	.12	.08	1.13	.26	.05
	Enjoyment	.23	.09	.15	2.55	.01	.12

^a^*B*: unstandardized coefficients.

^b^*sr*: semipartial correlation.

^c^*F*_2,176_=3.99, *P*=.02, adjusted *R*^2^=.033.

^d^*F*_change3,173_=88.95, *P*<.001, *R*^2^_change_=.58.

^e^PBC: perceived behavioral control.

^f^*F*_change3,170_=3.54, *P*=.02, *R*^2^_change_=.02.

## Discussion

### Principal Findings

The three TPB variables (attitudes, subjective norms, and PBC) were significant predictors of intention to play *Pokemon Go* while walking in both studies. Although attitudes and subjective norms were the positive predictors of intention to play the mobile game, PBC had negative impact on intention (ie, the less they feel in control, the more they play the mobile game while walking). Automaticity tendency, immersion, and enjoyment were significant predictors in study 1, whereas enjoyment was the only significant predictor in study 2.

Compared with the long tradition of research on people’s perception, attitudes, and behaviors affected by newspaper, magazines, radio, television, and the Internet, we have relatively little understanding of the psychological and behavioral impact of mobile media. There has been a drastic increase in pedestrian injuries while using mobile phone, but little research has been conducted to explain individuals’ motivations to use mobile devices while walking. This research identifies the predictors of intention to play a popular mobile game, *Pokemon Go*, while walking, by combining two theoretical approaches: TPB in health communication and recent findings related to behavioral orientations regarding mobile devices use in the field of media effect. This study addresses a very timely issue by recruiting the users who play *Pokemon Go*, one of the most popular augmented reality games that brought up significant concerns about safety issues both for pedestrians and drivers. Findings from this study contribute to expanding the scope of TPB to an emerging area in health communication—mobile device use and pedestrian safety, in particular playing a mobile game while walking.

Overall, the model we tested explained 41% of people’s intention to play a mobile game while walking in study 1 and 63% of people’s intention to play a mobile game while walking in study 2, with attitude toward playing a mobile game while walking emerging as the strongest predictor, followed by subjective norms. Both in study 1 and study 2, the three TPB components significantly predicted one’s intention to play a mobile game while walking. The three TPB components explained 37.6% in study 1 and 58% in study 2 of behavioral intention to play a mobile game in this study. The results indicated that attitude and subjective norms were positive significant predictors of intention to play a mobile game while walking, whereas PBC was a negative significant predictor of intention to play a mobile game while walking. The more they thought that playing the mobile game while walking was positive and beneficial, the greater their intention to play was; the more they believed that others would like them to play the mobile game while walking, the greater their intention to play was. However, the less they felt that they had control over playing it while walking, the greater their intention to play was.

The results demonstrated the utility of TPB in the context of mobile-related health behavior. TPB has been applied to many health-related behaviors including behaviors that cause public safety issues such as distracted driving [1,7,27]. Only few attempts have been made to apply the theory to mobile media usage [3,47], and this study is one of the very few studies that examined predictors of playing a mobile game while walking. Theoretically, our results demonstrated that attitudes, normative beliefs, and perceived control beliefs are the three main predictors to explain this new behavior. The accessibility and mobility of new media technology is new, but the underlying psychological motivations that drive individuals’ mobile media use while walking share commonality with other behavioral decision-making. TPB assumes that volitional behaviors will be best predicted from behavioral intention [19]. Playing a mobile game is a volitional behavior as people often engage in the behavior voluntarily and without coercion. Future research should examine the association between intention to play a mobile game and actual behavior to confirm the primary assumption of TPB. Overall, findings from this study demonstrate the predictive and explanatory power of TPB in a new context that has a significant implication for public safety—mobile game play while walking.

Especially, the significant effect of normative beliefs is thought-provoking in that previous studies pointed out that mundane tasks such as road crossing were less likely to be influenced by normative beliefs [47,48]. Perhaps, playing mobile games while walking is still considered to be uncommon, and individuals adhere to social norms (ie, perceived prevalence and approval) to evaluate the behavior. In addition, the popularity of *Pokemon Go* and recent media coverage on its safety issues (eg, news covering people who got injured while distracted by the game) may have enhanced the effects of social norms related to this particular game. For campaign message designers, findings from this study suggest that campaign messages should incorporate normative information to influence people’s mobile-related behaviors, including mobile game play while walking.

Contrary to the hypothesis 3, there was a negative association between individuals’ PBC and behavioral intention to play the game. In fact, previous research pointed out that a lesser degree of PBC leads to greater behavioral intention [49] when a behavior in question is socially undesirable. In other words, individuals often perform an undesirable behavior because they find it irresistible. Results from this study suggest that playing a mobile game while walking could be regarded as an undesirable as well as an addictive behavior. Players are aware of the fact that playing while walking may cause potential health threats to themselves as well as others, but they feel little control over this habitual behavior.

Further supporting this point, the feeling of enjoyment was a positive significant predictor of intention to play a mobile game in both study 1 and study 2. People who felt greater enjoyment were more likely to intend to play the game while walking than those who felt less enjoyment. This finding is consistent with prior literature on the effect of enjoyment on intention to play a Web-based game [35]. Wu and Liu found that enjoyment had a direct effect as well as an indirect effect via attitude on intention to play a Web-based game [35].

This study also found that automaticity and immersion were significant predictors of playing the mobile game in study 1. The automaticity and immersion factors increased by 4.4% of the amount of explained variances in intention to play a mobile game in addition to enjoyment factor in study 1. As college students played the mobile game more habitually and impulsively, they were more likely to play it while walking. This implies that people who have a tendency to play mobile game without intending to do so are more susceptible to perform such behaviors, which is consistent with prior literature’s findings on habitual mobile media use [33]. In contrast, immersion was a negative predictor of intention to play, suggesting that immersive players are less likely to play a mobile game while walking. Given that immersive users tend to play the mobile game with greater attention and often feel completely absorbed in playing the mobile game, they might be more reluctant to play it in a distracted setting. Although texting frequency was predicted only by automaticity, not immersion in a prior study [33], this study found that mobile game play while walking was negatively associated with immersion, perhaps because playing a mobile game requires a significant amount of time and attentional resource to be fully absorbed.

Our correlation results reported in Tables 3 and 4 provide further information to understand these ostensibly contradictory tendencies: automaticity and immersion. Although PBC was negatively correlated with automaticity, its correlation with immersion was not significant in both studies. Individuals who feel greater control over playing a mobile game while walking are less likely to play it impulsively and habitually. Instead, they could realize that they start playing a mobile game while walking, and probably stop themselves from doing it. However, this does not necessarily mean that they are less immersed while playing a mobile game. Regardless of their perceived control over the behavior, users can feel absorbed in playing a mobile game while walking.

In fact, it has to be noted that individuals’ levels of automaticity and immersion were significantly, and positively, correlated with each other in both studies. Bayer et al also found that these two tendencies are highly correlated in texting behavior—individuals who habitually text also do so with more immersion [33]. Our results replicated this finding and suggest that individuals who play a mobile game while walking do so at both high and low levels of attention. In other words, for the most respondents in our sample, they start playing a mobile game without meaning to do it, but at the same time, they get lost in the moment while they are playing it while walking.

Yet, these behavioral tendencies were not significant predictors of the intention of playing a mobile game while walking in study 2. In study 2, enjoyment was the only additional predictor of intention to play apart from the three components of TPB. It should be noted that there were differences in terms of sample characteristics between study 1 and study 2. The participants in study 1 were recruited from a university, whereas study 2 participants were recruited through MTurk, the crowdsourcing website that is known to be more representative of the US population. In addition, a post-hoc analysis using an analysis of variance (ANOVA) revealed that there was a significant difference in the degree of mobile phone addiction between study 1 (mean 5.07 [SD 0.99]) and study 2 (mean 4.74 [SD 1.26]). Participants in study 1 reported greater levels of mobile phone addiction (*F*_1,439_=9.27, *P*=.002) than those in study 2. In short, study 1 participants were in general younger, more homogeneous in their education, and more addicted to mobile phone use.

For these college students, their intention to play a mobile game can be significantly explained by their behavioral tendencies, because playing a mobile game is more than mere enjoyment to them. For addicted users, their habitual and impulsive tendency of playing a mobile game while walking would be an additional motivational factor that leads to greater intention to play. In addition, younger users’ attention is more likely hijacked by immersive media such as video games [50], and the degree of immersion that they experience from the media may have greater impact on how much they want to be engaged in the media use. In contrast, for older users who are less addicted to their mobile phones, whether they enjoy playing the mobile game is a more important predictor, regardless of their behavioral tendencies.

This finding suggests that playing a mobile game such as *Pokemon Go* has an addictive component, especially for college students. Individuals who are more likely to play a mobile game while walking also pay less attention and think less while playing the game, habitually performing the behavior. Video game addiction and its detrimental effects on well-being [51,52] are well known, but mobile game addiction has been only recently highlighted by researchers [53]. An augmented reality game such as *Pokemon Go* may provide even stronger senses of presence and flow [54] because of its capability of seamlessly blending the virtual world into the real world, leading to more habitual and addictive game play. Health campaigns targeting mobile users should point out the possibility of addiction and incorporate it into their prevention messages regarding playing a mobile game while walking.

In addition, there was a considerable difference in the amount of explained variances in intention: study 1 explained 41%, whereas study 2 explained 63% of variance in their intention to play. Our model focused on attitudes, subjective norms, and PBC as main predictors, which did not address individuals’ digital media use patterns in general, including the level of addiction discussed above. College students in study 1 may have already developed their own digital media preference and could have been more experienced in mobile game play, whereas older users in study 2 may have been less influenced by these factors. Future research may benefit from identifying more media use variables for predicting college students’ mobile game play while walking.

### Practical Implications

The studies’ findings have implications for health promotion practice and policy. First, health practitioners and designers should incorporate normative information in their campaign messages to amend people’s false beliefs and optimistic biases, and thus change such habitual and addictive behaviors in a desirable way. Second, inattention is one of prevalent causal factors of road incidents [10]. When playing mobile-based augmented reality exergames such as *Pokemon Go* while walking, running, or cycling, pedestrians would focus more on their screens and thus be less aware of their surroundings. Therefore, augmented reality functions and technologies should incorporate realistic features to increase the special and temporal awareness to avoid potential risks and motivate physical activity [55].

### Limitations

This study investigated playing a mobile game as the only target behavior. However, it seems significant to examine other mobile usage behaviors (eg, social media use such as Snapchat, listening to music, or taking pictures or videos while walking), given that they may trigger different types of safety issues from playing a mobile game. In addition, it is still unclear how different predictors of TPB would be applied to a different set of mobile behaviors. For instance, subjective norms were not a significant predictor for crossing streets while listening to music or texting [47], whereas findings from this study indicated that normative influence was associated with playing a mobile game while walking in our model. Thus, future studies ought to investigate different psychological factors associated with different scenarios of distracted walking or pedestrian safety issues.

By showing the significant effect of individuals’ consciousness tendency on behavioral intention to play mobile games while walking, this study suggests the need for future studies on habitual or addictive mobile media use. Individuals who habitually use mobile media while walking, without meaning to do it, might be more likely to experience serious health threats such as accidents. Therefore, future studies ought to investigate some predictors of the unconscious media use, including personality and situational factors, and further analyze how these factors are associated with mobile game play in unsafe settings.

Our sample size was not big, limiting the generalizability of our results. After selecting only those who played *Pokemon Go*, we had 262 respondents for study 1 and 179 respondents for study 2 who completed the survey. In addition, respondents’ actual behavior or experience of being injured by distracted walking was not addressed in this study. Future studies should test the effect of predictors of TPB on individuals’ actual mobile use behavior while walking, as well as the effect of personal experiences. Given the cross-sectional design of this study, causal claims could not be made. On the basis of TPB, it was assumed that three predictors precede people’s intention to play a mobile game while walking. However, it is also likely that the temporal order of these two variables could be reversed. In future research, a longitudinal design or experimental method should be employed to explore the causal relationship between three TPB predictors and intention.

## Appendix

Multimedia Appendix 1 Survey questionnaire.

## Abbreviations

ANOVA: analysis of variance
MTurk: Amazon Mechanical Turk
PBC: perceived behavioral control
SD: standard deviation
SE: standard error
TRA: theory of reasoned action
TPB: theory of planned behavior
