# Correction: Predictors of Playing Augmented Reality Mobile Games While Walking Based on the Theory of Planned Behavior: Web-Based Survey

**DOI:** 10.2196/49937

**Published:** 2023-06-19

**Authors:** Hyeseung Elizabeth Koh, Jeeyun Oh, Michael Mackert

**Affiliations:** 1 The Center for Health Communication Moody College of Communication The University of Texas at Austin Austin, TX United States; 2 Stan Richards School of Advertising and Public Relations Moody College of Communication The University of Texas at Austin Austin, TX United States; 3 Department of Population Health Dell Medical School The University of Texas at Austin Austin, TX United States; 4 School of Public Health The University of Texas Health Science Center at Houston Houston, TX United States

In "Predictors of Playing Augmented Reality Mobile Games While Walking Based on the Theory of Planned Behavior: Web-Based Survey" (JMIR Mhealth Uhealth 2017;5(12):e191) the authors noted one error.

In Table 6, the results for the factor “Enjoyment” in the third block were shifted one column to the right. The original table can be seen in Multimedia Appendix 1. This has been changed to read as follows:

**Table 6:** Regression results for intention to play a mobile game while walking in study 2 (N=197).

Predictors		*B*	Standard error (SE)	Beta	*t*	*P*	*sr*
**Third Block**							
	Enjoyment	.23	.09	.15	2.55	.01	.12

The correction will appear in the online version of the paper on the JMIR Publications website on June 19, 2023, together with the publication of this correction notice. Because this was made after submission to PubMed, PubMed Central, and other full-text repositories, the corrected article has also been resubmitted to those repositories.

## Appendix

Multimedia Appendix 1 Original published version of “Table 6. Regression results for intention to play a mobile game while walking in study 2 (N=197).”.

