# Supplementary material for: Predictors of Playing Augmented Reality Mobile Games While Walking Based on the Theory of Planned Behavior: Web-Based Survey
Source: JMIR Mhealth Uhealth. 2017 Dec 11;5(12):e191. doi: 10.2196/mhealth.8470 (PMC5742645; doi:10.2196/mhealth.8470)
Supplement: Multimedia Appendix 1 [file mhealth_v5i12e191_app1.pdf]

Please answer the following questions to the best of your ability. There is no right or wrong answer. You do not have to answer any questions that you feel uncomfortable answering.

1. For me, playing Pokemon Go while walking would be...

	1 (1)	2 (2)	3 (3)	4 (4)	5 (5)	6 (6)	7 (7)
Bad:Good	❍	❍	❍	❍	❍	❍	❍
Foolish:Wise	❍	❍	❍	❍	❍	❍	❍
Negative:Positive	❍	❍	❍	❍	❍	❍	❍
Unpleasant:Pleasant	❍	❍	❍	❍	❍	❍	❍
Harmful:Beneficial	❍	❍	❍	❍	❍	❍	❍
Unenjoyable:Enjoyable	❍	❍	❍	❍	❍	❍	❍
Unfavorable:Favorable	❍	❍	❍	❍	❍	❍	❍

2. Please indicate how much you agree with the following statements. Your answer can range from strongly disagree to strongly agree.

	Strongly Disagree (1)	Disagree (2)	Somewhat Disagree (3)	Neither Agree nor Disagree (4)	Somewhat Agree (5)	Agree (6)	[illegible]
1. Those people who are important to me would approve of me playing Pokemon Go while walking.	❍	❍	❍	❍	❍	❍	
2. Those people who are important to me would want me to play Pokemon Go while walking.	❍	❍	❍	❍	❍	❍	
3. Those people who are important to me would think I should play Pokemon Go while walking.	❍	❍	❍	❍	❍	❍	
4. Those people who are important to me wish that I play Pokemon Go while walking.	❍	❍	❍	❍	❍	❍	
5. A number of my friends are playing Pokemon Go while walking.	❍	❍	❍	❍	❍	❍	
6. A number of my friends are about to play Pokemon Go while walking.	❍	❍	❍	❍	❍	❍	
7. A number of friends have played Pokemon Go while walking.	❍	❍	❍	❍	❍	❍	

3. Please click the button that best represents your idea.

	Strongly Disagree (1)	Disagree (2)	Somewhat Disagree (3)	Neither Agree nor Disagree (4)	Somewhat Agree (5)	Agree (6)	[illegible]
1. Whether or not I play Pokemon Go while walking is entirely up to me.	❍	❍	❍	❍	❍	❍	

	Absolutely No Control (1)	Very Little Control (2)	Little Control (3)	Just Right (4)	Somewhat Control (5)	A Lot Control (6)	[illegible]
2. How much personal control do you feel you have over a behavior, playing Pokemon Go while walking?	❍	❍	❍	❍	❍	❍	

	Not At All (1)	Very Little Control (2)	Little Control (3)	Just Right (4)	Somewhat Control (5)	A Lot Control (6)	[illegible]
3. How much do you feel that whether you play Pokemon Go while walking is beyond your control?	❍	❍	❍	❍	❍	❍	

4. When I play Pokemon Go while walking,

	Not at all (1)	Slightly (2)	Somewhat (3)	Completely Mostly (4)	Completely (5)
1. I do it without thinking.	❍	❍	❍	❍	❍
2. I begin doing it before I realize I'm doing it.	❍	❍	❍	❍	❍
3. I do it without meaning to do it.	❍	❍	❍	❍	❍
4. I find it hard to stop myself from doing it.	❍	❍	❍	❍	❍

5. When I play Pokemon Go while walking,

	Not at all (1)	Slightly (2)	Somewhat (3)	Completely Mostly (4)	Completely (5)
1. I'm absorbed in doing it.	❍	❍	❍	❍	❍
2. My eyes are fixed on doing it.	❍	❍	❍	❍	❍
3. It takes me away from what is going on around me.	❍	❍	❍	❍	❍
4. I get lost in the moment while I'm doing it.	❍	❍	❍	❍	❍

6. Please indicate how much you agree with the following statements. Your answer can range from strongly disagree to strongly agree.

	Strongly Disagree (1)	Disagree (2)	Somewhat Disagree (3)	Neither Agree nor Disagree (4)	Somewhat Agree (5)	Agree (6)
1. Playing Pokemon Go is enjoyable.	❍	❍	❍	❍	❍	❍
2. Playing Pokemon Go is exciting.	❍	❍	❍	❍	❍	❍
3. Playing Pokemon Go is interesting.	❍	❍	❍	❍	❍	❍
4. Playing Pokemon Go is boring.	❍	❍	❍	❍	❍	❍

7. For the following questions, please click the button that you feel best represents your response.

	Strongly Disagree (1)	Disagree (2)	Somewhat Disagree (3)	Neither Agree nor Disagree (4)	Somewhat Agree (5)	Agree (6)
1. I plan to play Pokemon Go while walking.	❍	❍	❍	❍	❍	❍
2. I intend to play Pokemon Go while walking.	❍	❍	❍	❍	❍	❍
3. It is likely that I will play Pokemon Go while walking.	❍	❍	❍	❍	❍	❍

8. Please click the button that best represents your thought.

	Strongly Disagree (1)	Disagree (2)	Somewhat Disagree (3)	Neither Agree nor Disagree (4)	Somewhat Agree (5)	Ag
1. I often use my cell phone for no particular reason.	❍	❍	❍	❍	❍	
2. I feel connected to others when I am using my cell phone.	❍	❍	❍	❍	❍	
3. I am concerned that I rely too much on my cell phone.	❍	❍	❍	❍	❍	
4. I keep checking my cell phone for messages or calls.	❍	❍	❍	❍	❍	
5. I feel anxious when I am unable to use my cell phone.	❍	❍	❍	❍	❍	
6. The thought of being without my cell phone makes me feel distressed.	❍	❍	❍	❍	❍	
7. I get excited when I hear my cell phone ring.	❍	❍	❍	❍	❍	
8. I get excited when I receive a text message.	❍	❍	❍	❍	❍	

9. What is your age?   For example, if you are 21 years-old, put the number 21 below.

10. When were you born? For example, if you were born in 1992, put the number 1992 below.

11. What is your sex?
- ❍ Male (0)
- ❍ Female (1)

12. Your ethnicity is
- ❍ Caucasian (1)
- ❍ Asian / Asian American (2)
- ❍ Hispanic / Hispanic American (3)
- ❍ African American (4)
- ❍ Native American (5)
- ❍ Pacific Islander (6)
- ❍ Others (7)

[The following questions are only for Study 2 – Mturk]

13. What is the highest level of education you have completed so far?
- Less than high school (1)
- High school/GED (2)
- Some college (3)
- 2 year college degree (Associate) (4)
- 4 year college degree (BA/BS) (5)
- Professional degree (MD/JD) (6)
- Master's degree (7)
- Doctoral degree (8)

14. What is your total household income last year (before tax)?
- Less than $25,000 (1)
- $25,000 to $49,999 (2)
- $50,000 to $74,999 (3)
- $75,000 to $99,999 (4)
- $100,000 or more (5)
