# Supplementary material for: Correction: Predictors of Playing Augmented Reality Mobile Games While Walking Based on the Theory of Planned Behavior: Web-Based Survey
Source: JMIR Mhealth Uhealth. 2023 Jun 19;11:e49937. doi: 10.2196/49937 (PMC10337319; doi:10.2196/49937)
Supplement: Multimedia Appendix 1 [file mhealth_v11i1e49937_app1.docx]

**Multimedia Appendix 1**: Original published version of “Table 6. Regression results for intention to play a mobile game while walking in study 2 (N=197).”.

Table 6. Regression results for intention to play a mobile game while walking in study 2 (N=197).

| Predictors | | *B*^a^ | Standard error (SE) | Beta | *t* (df=195) | *P* value | *sr*^b^ |
| --- | --- | --- | --- | --- | --- | --- | --- |
| **First block**^c^ | |  |  |  |  |  |  |
|  | Age | −.02 | .02 | −.07 | −0.91 | .36 | −.07 |
|  | Gender | −.76 | .30 | −.19 | −2.59 | .01 | −.19 |
| **Second block**^d^ | |  |  |  |  |  |  |
|  | Attitude | .73 | .08 | .53 | 8.73 | <.001 | .41 |
|  | Subjective norms | .46 | .09 | .33 | 5.28 | <.001 | .25 |
|  | PBC^e^ | −.29 | .09 | −.16 | −3.29 | .001 | −.15 |
| **Third block**^f^ | |  |  |  |  |  |  |
|  | Attitude | .60 | .09 | .44 | 6.56 | <.001 | .30 |
|  | Subjective norms | .44 | .09 | .32 | 5.06 | <.001 | .23 |
|  | PBC^c^ | −.35 | .10 | −.19 | −3.63 | <.001 | −.17 |
|  | Automaticity | −.06 | .12 | −.04 | −0.54 | .59 | −.03 |
|  | Immersion | .14 | .12 | .08 | 1.13 | .26 | .05 |
|  | Enjoyment |  | .23 | .09 | .15 | 2.55 | .01 |

^a^*B*: unstandardized coefficients.

^b^*sr*: semipartial correlation.

^c^*F*_2,176_=3.99, *P*=.02, adjusted *R*^2^=.033.

^d^*F_change_* _3,173_=88.95, *P*<.001, *R*^2^*_change_*=.58.

^e^PBC: perceived behavioral control.

^f^*F_change_* _3,170_=3.54, *P*=.02, *R*^2^*_change_*=.02.
